# Influence of dialysate temperature on creatinine peritoneal clearance in peritoneal dialysis patients: a randomized trial

**DOI:** 10.1186/s12882-020-02113-z

**Published:** 2020-10-27

**Authors:** Francesco Fontana, Chiara Torelli, Silvia Giovanella, Giulia Ligabue, Gaetano Alfano, Karin Gerritsen, Rafael Selgas, Gianni Cappelli

**Affiliations:** 1 Surgical, medical and dental department of morphological sciences, Section of Nephrology, University of Modena and Reggio Emilia, Modena, Italy; 2 Nephrology and Dialysis Unit, Azienda Ospedaliero-Universitaria di Modena, Modena, Italy; 3 Department of Nephrology and Hypertension, University Medical Center Utrecht, Utrecht, The Netherlands; 4 Nephrology Department, Hospital Universitario La Paz, IdiPAZ, Universidad Autonoma de Madrid, REDinREN, IRSIN, Madrid, Spain

**Keywords:** Dialysate temperature, Peritoneal dialysis, Peritoneal clearance

## Abstract

**Background:**

Patients on continuous ambulatory peritoneal dialysis (PD) are encouraged to warm dialysate to 37 °C before peritoneal infusion; main international PD guidelines do not provide specific recommendation, and patients generally warm dialysate batches partially or do not warm them at all. Warming of dialysate is a time-consuming procedure, not free from potential risks (i.e. degradation of glucose), and should be justified by a clear clinical benefit.

**Methods:**

We designed a single blind randomized controlled trial where 18 stable PD patients were randomized to receive a peritoneal equilibration test either with dialysate at a controlled temperature of 37 °C (intervention group) or with dialysate warmed with conventional methods (control group). Primary end-point was a higher peritoneal creatinine clearance in patients in the intervention group.

**Results:**

Patients in the intervention group did not show a significantly higher peritoneal creatinine clearance when compared to the control group (6.38 ± 0.52 ml/min vs 5.65 ± 0.37 ml/min, *p* = 0.2682). Similar results were obtained for urea peritoneal clearance, mass transfer area coefficient of creatinine and urea. There were no significant differences in total abdominal discomfort questionnaire score, blood pressure and body temperature between the two groups.

**Conclusions:**

Using peritoneal dialysate at different temperatures without causing significant side effects to patients appears feasible. We report a lack of benefit of warming peritoneal dialysate to 37 °C on peritoneal clearances; future PD guidelines should not reinforce this recommendation.

**Trial registration:**

NCT04302649, ClinicalTrials.gov; date of registration 10/3/2020 (retrospectively registered).

**Supplementary Information:**

The online version contains supplementary material available at 10.1186/s12882-020-02113-z.

## Background

Peritoneal dialysis (PD) currently represents the main choice for home renal replacement treatment for patients with end stage renal disease. One of the limitations of PD technique is represented by the difficulty in achieving target dialytic clearances and PD adequacy for some patients, especially with increasing PD vintage [1–3]. A potentially relevant issue in PD clearances is the effect of dialysate temperature on depuration. Indeed, it is common for clinicians to advise patients in Continuous Ambulatory Peritoneal Dialysis (CAPD) to warm the dialysate before infusion into the peritoneal cavity, with different methods (microwave oven, warming cabin, warming pad). Nevertheless, main international PD guidelines do not provide specific recommendations on this topic [4, 5]. Only guidelines from the British Columbia Renal Agency (Canada) [6] dedicate a specific chapter to the temperature of dialysate, recommending its warming to 37 °C before peritoneal infusion, mainly in order to avoid an “uncomfortable lowering of body temperature”. On the other hand, warming of PD batches could lead to hot spots formation inside the batch, especially with microwaves, and to degradation of glucose leading to the formation of toxic glucose degradation products (GDPs) [7]. Also, notable differences in room temperature exist according to geographical latitude and year season, and there are no clear and detailed reports in the literature regarding intolerable effects of the infusion of dialysate at room temperature. It must be acknowledged that it is common practice for patients to warm dialysate batches only partially or not to warm them at all. Moreover, warming pads that are most commonly used by CAPD patients do not effectively warm the dialysate up to 37 °C. In our center we recently observed that average dialysate temperature at infusion was 31.1 °C, even if the pad was calibrated to 37 °C [unpublished data]. With respect to the effects on toxins clearances through the peritoneal membrane, a higher dialysate temperature could theoretically favor vasodilation of peritoneal membrane microcirculation, potentially increasing the passage of substances. Severe microcirculatory dysfunction has been reported in PD patients [8] and any intervention designed to ameliorate microcirculatory flow at peritoneal level could be beneficial. Surprisingly, reports regarding the effects of dialysate temperature on peritoneal clearances in PD in humans are surprisingly scarce. In 1967 Gross et al. [9] reported an increase in the exchange of substances between peritoneal fluid and blood upon warming of the PD fluid to 37 °C (compared to 20 °C) in a patient treated with intermittent peritoneal dialysis; the increase in urea clearance with the 37 °C solution was 35% on average. In contrast, Indraprasit et al. [10] did not encounter differences in peritoneal creatinine clearance utilizing dialysate at room temperature (27–31 °C) and warmed at 37 °C in a group of 18 patients in PD. Confirmation of the effects of dialysate temperature on peritoneal clearances would be of great interest in order to maximize the depurative potential of PD and to justify patients’ effort to warm the batches.

In order to determine the real effects of dialysate temperature on peritoneal clearances and transport characteristics, abdominal discomfort and vital signs, we designed a randomized controlled trial comparing two strategies of peritoneal dialysate warming.

## Methods

### Study design and participants

**Fig. 1:**
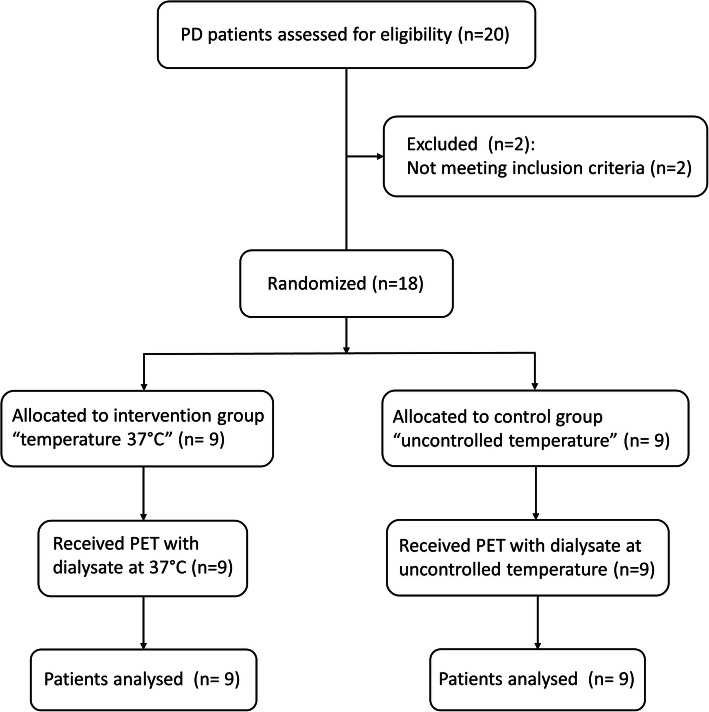
Study flow diagram

Eighteen PD patients, both in CAPD and automated PD, in regular follow-up at the Nephrology Unit of the University Hospital of Modena, were randomized to receive a single dialysis exchange, either with dialysate at a controlled temperature of 37 °C (intervention group) or with dialysate at warmed with conventional methods at uncontrolled temperature (control group). See Fig. 1 for a participants flow diagram. Randomization was generated through the use of the Random Allocation software [11].

Primary end-point of our study was peritoneal creatinine clearance. Secondary end-points were: peritoneal urea clearance, creatinine and urea dialysate/plasma ratio (D/P), creatinine and urea mass transfer area coefficient (MTAC), abdominal discomfort, blood pressure and body temperature.

A power analysis was performed while designing the study using the few data available in the literature [9, 10]; setting the alpha-error level at 0.05 for a 2-tailed t-test with a statistical power of 95% (beta-error 0.05) the estimated sample needed was 14 patients (7 per group). We thus decided to enroll 9 PD patients per group. Patients were recruited from September to December 2018; the trial ended when the pre-specified number of enrolled patients was achieved. The study was conducted in accordance with the Helsinki Declaration of 1975 (as revised in 2008) and was approved by the ethical committee “Comitato Etico dell’Area Vasta Emilia Nord” of the “Azienda Ospedaliero Universitaria di Modena” (protocol number: AOU:0018014/18). The trial was registered at ClinicalTrials.gov (NCT number: NCT04302649). All study participants received adequate information before enrollment and signed informed consent. The study adheres to CONSORT guidelines for reporting clinical trials (see supplementary material).

Inclusion criteria were: age > 18 years and ability to give informed consent, PD dialysis treatment (either continuous ambulatory peritoneal dialysis or automated peritoneal dialysis), PD vintage of more than 3 months, absence of signs of active acute systemic or localized infections at least 4 weeks apart from the trial (i.e. peritonitis). Pregnant women were excluded from the study.

### Laboratory measurements

Creatinine and urea were measured in plasma, peritoneal fluid and urine at the Central Laboratory of “Policlinico di Modena” with the Jaffé and colorimetric methods, and expressed in mg/dl.

Peritoneal clearances of creatinine and urea were calculated with the following formula:

$$ Cx=\left[ Dx\right]\times dialysate\ volume/\left[ Px\right]/ 240 $$

where Cx represents clearance of creatinine or urea expressed in ml/min, [Dx] represents the concentration of creatinine or urea in dialysate at the end of the exchange (4 h) expressed in mg/dl, dialysate volume represents the total volume drained at the end of the exchange (4 h), [Px] represents the concentration of creatinine or urea in plasma after 2 h from the beginning of the exchange expressed in mg/dl and 240 represents minutes contained in the 4 h of the exchange.

Data regarding D/P and dialysate volume were also reported separately.

Mass transfer area coefficients (MTAC) for creatinine and urea were calculated with the RenalSoft software (converted from the PD Adequest software [12]) from Baxter Healthcare, Deerfield, IL, U.S.A. Correction for plasmatic water concentration was not added, since the main purpose was to compare MTACs from the intervention and control group and not to obtain absolute data. MTAC is related to the restriction coefficient for a specific solute through a double logarithmic scale. Variations of the MTAC of a small solute are mainly caused by variation in the vascular surface area, and thus the number of available pores. A rise in MTAC indicates an increase in the number of available pores and vice versa [13].

Urinary clearances of creatinine and urea were calculated with the following formula:

$$ Cx=\left[ Ux\right]\times urine\ volume/\left[ Px\right]/ 240 $$

where Cx represents clearance of creatinine or urea expressed in ml/min, [Ux] represents the concentration of creatinine or urea in urine at the end of the exchange (4 h) expressed in mg/dl, urine volume represents the total volume collected at the end of the exchange (4 h), [Px] represents the concentration of creatinine or urea in plasma after 2 h from the beginning of the exchange expressed in mg/dl and 240 represents minutes contained in the 4 h of the exchange. Patients were required to empty their bladder before the exchange.

Blood pressure was measured hourly during the exchange with an automatic sphygmomanometer and expressed in mmHg.

Body temperature was measured hourly during the exchange with an auricular thermometer and expressed in °C.

Abdominal discomfort was monitored through specific questions to the patient hourly during the exchange and through the administration of a specific questionnaire at the end of the exchange. The abdominal discomfort questionnaire was adapted from Figueiredo et al. [14], and contained 7 specific questions regarding the appearance of the following symptoms during or immediately after the exchange: chills, vomiting, abdominal pain, abdominal distension, constipation, diarrhea, loss of appetite. Every symptom was graded by the patient from 0 to 3 according to its intensity, and a total score for every patient was elaborated (range 0–21 points).

### Peritoneal exchange performance

All patients received an in-hospital 4 h Peritoneal Equilibration Test (PET) performed by nurses trained in the PD dialysis service of the Nephrology Unit of the University Hospital of Modena. The PET, originally proposed by Twardowski [15, 16], consists of a 4 h exchange with 2 l of peritoneal dialysate at a glucose concentration of 2.27% where plasma and dialysate concentration of creatinine and glucose are assessed at different time points during the test; the PET can be used to calculate peritoneal clearances and MTACs of creatinine and urea with the above mentioned formulas.

Patients received PET with dialysate at 37 °C or at uncontrolled temperature, according to randomization. In the intervention group, dialysate was warmed in a specific microwave oven calibrated to 37 °C and infusion temperature was confirmed to be 37 °C before infusion. In the control group, current practice was used (batch warming with a pad calibrated to 37 °C) and dialysate temperature was measured just before infusion.

All temperature measurements were performed with an infrared thermometer and expressed in °C.

A sample of peritoneal dialysate was drained and sent for analysis at the beginning of the test and at 2 and 4 h; the total amount of fluid drained at the end of the test was measured. A blood sample was drawn at 2 h from the beginning of the test and sent for analysis. The amount of urine produced during the 4 h test was measured and a sample sent for analysis.

Blood pressure and body temperature were monitored during the PET as previously described.

### Statistical analysis

Continuous data are presented as mean and standard error of the mean; discrete data are presented as proportions. Continuous variables were compared with Student’s t-test (two tailed). Proportions were compared with Fisher’s exact test. A *p* value lower than 0.05 was considered statistically significant. Statistical analysis was performed with GraphPad Prism software version 7.00 for Windows, GraphPad Software, La Jolla California USA, www.graphpad.com.

## Results

Characteristics of the intervention and control groups are reported in Table 1. As expected, there was a statistically significant difference in dialysate temperature between the intervention and control group (36.78 ± 0.38 °C vs 32.22 ± 0.26 °C, respectively, *p* < 0.0001). Other relevant patient characteristics were not significantly different between the two groups. Specifically, there were the same proportions of males, diabetics, hypertensive, high average peritoneal transporters and the two groups did not differ in age, PD vintage, residual urinary output, urinary creatinine clearance; moreover, main laboratory blood tests (including serum albumin and C-reactive protein) were similar between the two groups.

**Table 1:** Patient characteristics in the control (Uncontrolled T) and intervention (37 °C) groups

	Uncontrolled T (*n* = 9)	37 °C T (*n* = 9)	*p* value
**n. patients**	9,00	9,00	
**dialysate temperature (°C)**	32,22 ± 0,26	36,78 ± 0,38	< 0,0001
**males (percentage)**	78%	78%	ns
**diabetics (percentage)**	22%	56%	ns
**hypertensive (percentage)**	89%	89%	ns
**high average transporters (percentage)**	44%	44%	ns
**low average transporters (percentage)**	33%	44%	ns
**high transporters (percentage)**	0%	11%	ns
**low transporters (percentage)**	22%	0%	ns
**dialysis vintage (months)**	28,44 ± 9,11	25,56 ± 5,82	ns
**urinary creatinine clearance (ml/min)**	4,72 ± 2,76	6,32 ± 2,01	ns
**urine output (ml/4 h PET test)**	133,30 ± 72,65	188,90 ± 54,50	ns
**dialysate volume drained (ml)**	2267,00 ± 108,30	2156,00 ± 70,93	ns
**serum creatinine (mg/dl)**	9,53 ± 1,23	8,39 ± 0,79	ns
**serum urea (mg/dl)**	110,10 ± 8,55	118,00 ± 11,84	ns
**serum albumin (g/dl)**	3,70 ± 0,11	3,42 ± 0,13	ns
**serum hemoglobin (g/dl)**	11,60 ± 0,00	11,42 ± 0,36	ns
**serum c-reactive protein (mg/dl)**	0,29 ± 0,06	0,63 ± 0,28	ns

Data are presented as mean ± standard error of the mean (SEM) or percentages

*ns* non-significant

**Fig. 2:**
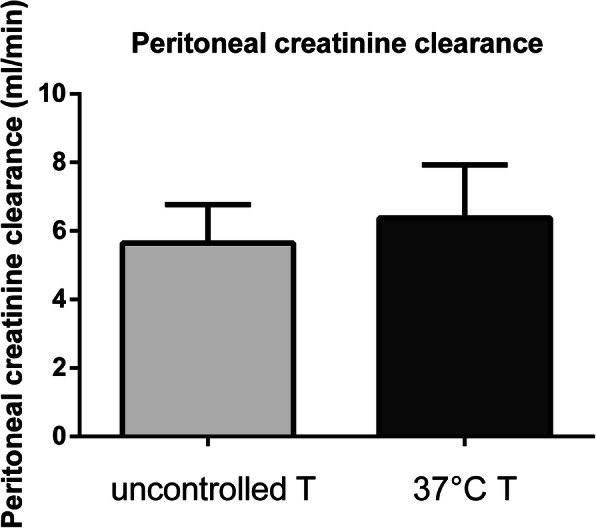
Peritoneal creatinine clearance in the uncontrolled dialysate temperature group (light grey bar) and 37° dialysate temperature group (dark grey bar)

**Fig. 3:**
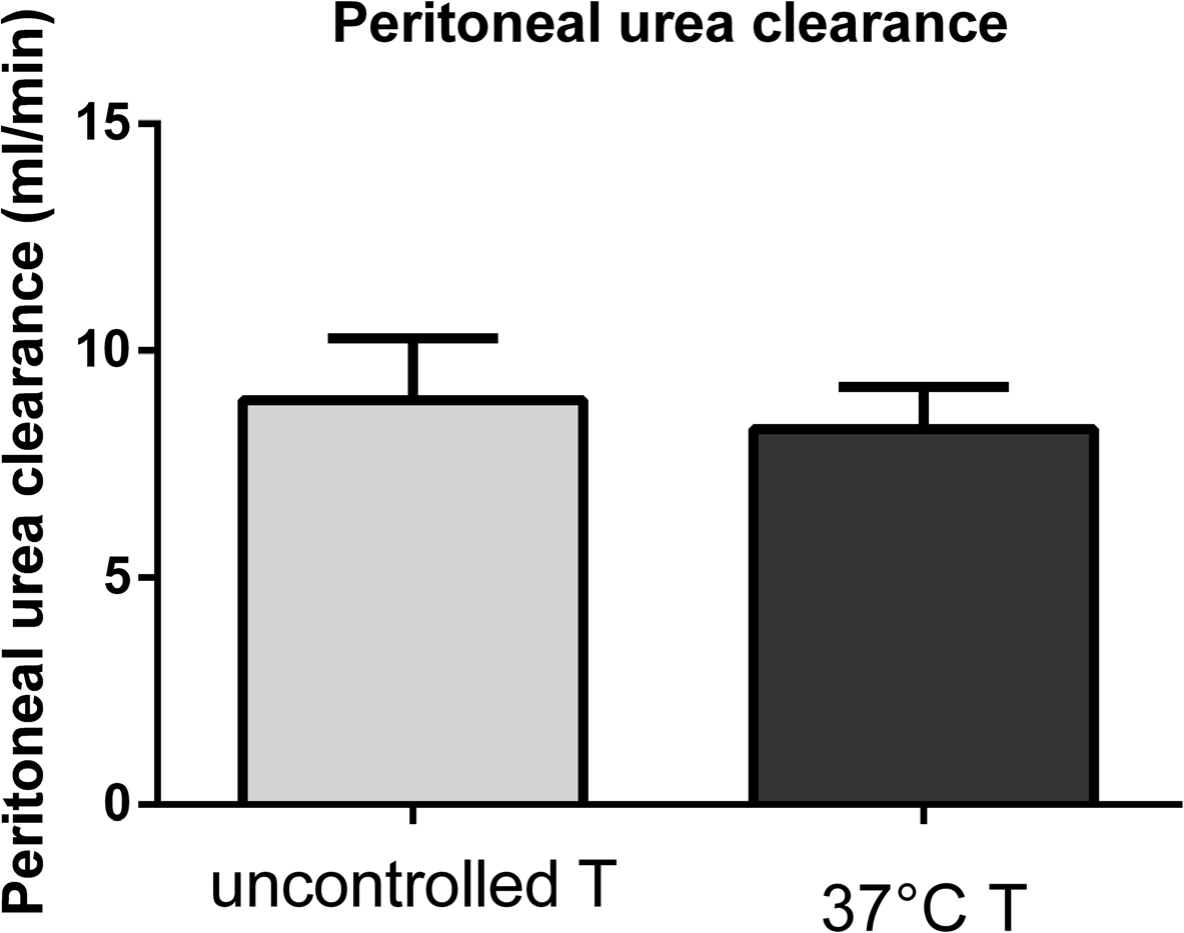
Peritoneal urea clearance in the uncontrolled dialysate temperature group (light grey bar) and 37° dialysate temperature group (dark grey bar)

**Fig. 4:**
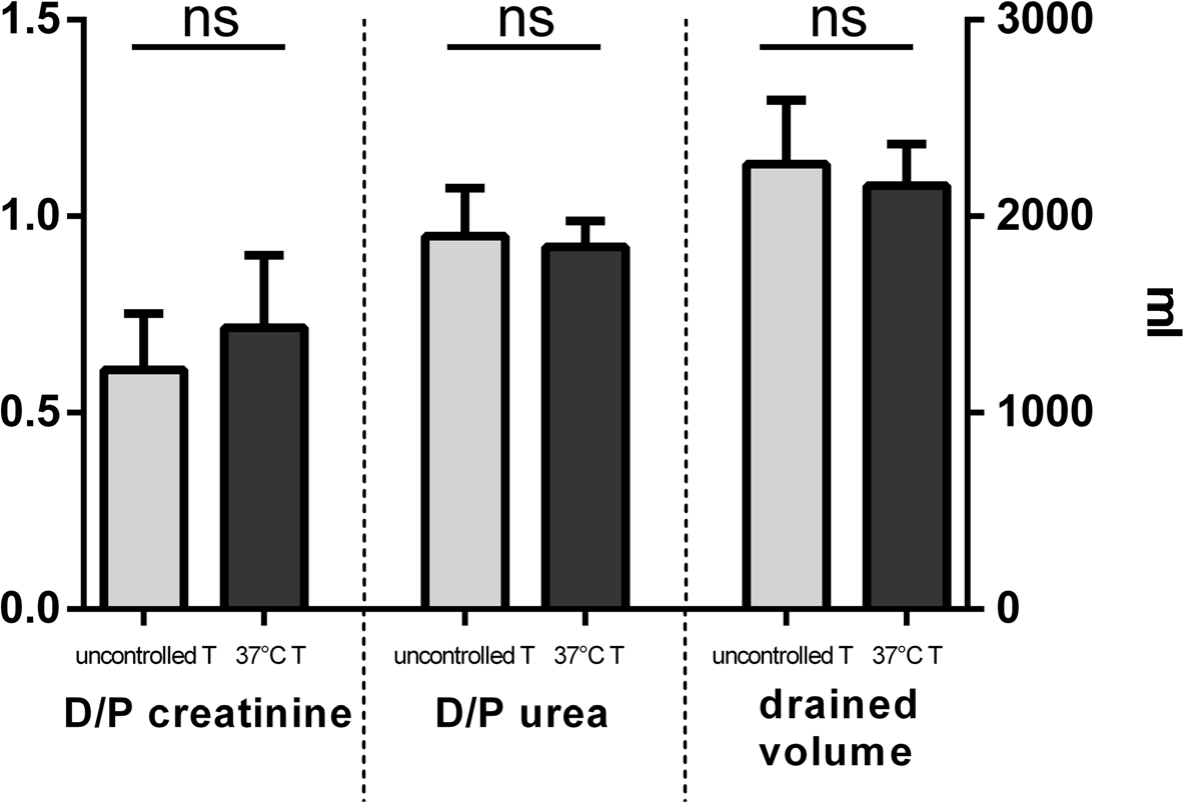
Dialysate to plasma ratio for creatinine and urea (left and middle panel, left y axis) and dialysate drained volume (right panel, right y axis) for the uncontrolled dialysate temperature group (light grey bar) and 37° dialysate temperature group (dark grey bar)

**Fig. 5:**
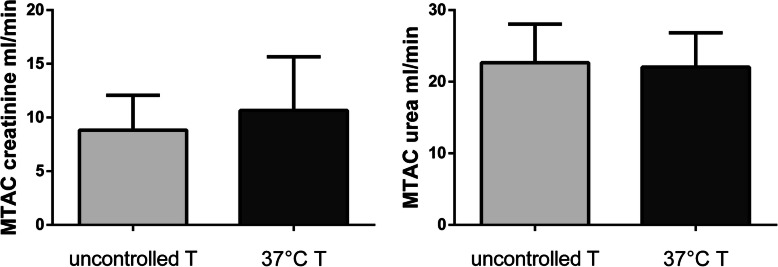
Peritoneal mass transfer urea coefficients (MTAC) for creatinine (left panel) and urea (right panel) in the uncontrolled dialysate temperature group (light grey bars) and 37° dialysate temperature group (dark grey bars)

Patients in the intervention group did not show a higher peritoneal creatinine clearance when compared to patients in the control group (6.38 *±* 0.52 ml/min vs 5.65 *±* 0.37 ml/min, *p* = 0.2682), as depicted in Fig. 2. In accordance with this result, we encountered no difference in urea peritoneal clearance (8.28 *±* 0.31 ml/min in the intervention group vs 8.92 *±* 0.45 ml/min in the control group, *p* = 0.2561); see Fig. 3. As a further confirmation, the two groups did not differ significantly for creatinine D/P (0,72 *±* 0.18 in the intervention group vs 0,61 *±* 0,14 in the control group, p = 0,1846), urea D/P (0.92 *±* 0.07 in the intervention group vs 0,95 *±* 0.12 ml/min in the control group, *p* = 0.5718) and drained dialysate volume (2156 *±* 212,8 ml in the intervention group vs 2267 *±* 325 ml in the control group, *p* = 0.4035) (Fig. 4). In addition, MTACs for creatinine and urea were not different between the two groups (MTAC creatinine 10.66 *±* 1.77 ml/min in the intervention group and 8.82 *±* 1.08 ml/min in the control group, *p* = 0.3781; MTAC urea 22.05 *±* 1.69 ml/min in the intervention group and 22.66 *±* 2.03 ml/min in the control group, *p* = 0.8199); see Fig. 5.

Patients showed very few abdominal symptoms and there were no significant differences in the total abdominal discomfort questionnaire score between the two groups (see Table 2). Blood pressure and body temperature at different time points were also comparable between the two treatment dialysate temperatures, as reported in Table 2.

**Table 2:** Secondary outcomes in the control (uncontrolled T) and intervention (37 °C T) groups

	Uncontrolled T	37 °C T	*p* value
**Abdominal discomfort questionnaire (points)**	0.38 ± 0.38	0.11 ± 0.11	ns
**Mean arterial pressure t0 (mmHg)**	102.8 ± 3.29	99.22 ± 5.01	ns
**Mean arterial pressure t1 (mmHg)**	97.33 ± 2.89	100.9 ± 3.86	ns
**Mean arterial pressure t2 (mmHg)**	97.93 ± 3.33	98.93 ± 4.09	ns
**Mean arterial pressure t3 (mmHg)**	98.41 ± 3.15	93.04 ± 3.11	ns
**Mean arterial pressure t4 (mmHg)**	97.37 ± 3.35	99.15 ± 5.78	ns
**Body temperature t0 (°C)**	35.56 ± 0.28	35.88 ± 0.32	ns
**Body temperature t1 (°C)**	35.57 ± 0.23	35.87 ± 0.26	ns
**Body temperature t2 (°C)**	35.67 ± 0.23	35.78 ± 0.17	ns
**Body temperature t3 (°C)**	35.86 ± 0.19	35.86 ± 0.17	ns
**Body temperature t4 (°C)**	35.86 ± 0.17	35.9 ± 0.17	ns

Continuous data are expressed as mean ± standard error of the mean (SEM). *ns* non-significant; t0, t1, t2, t3, t4 = measurements at 0, 1, 2, 3, 4 h from the beginning of the exchange

## Discussion

In the present study, we found no differences on peritoneal clearances of small molecules in a PET using dialysate at two different temperatures; also, no differences in abdominal discomfort and vital signs were noted.

Available literature data do not univocally determine the role of dialysate temperature with respect to peritoneal clearances, neither clear data exists about the occurrence of local or systemic side effects with different dialysate temperatures. Theoretically, an increase in temperature in the peritoneal cavity could lead to an increase in microcirculatory vasodilation at the local level, increasing the total pore area, in contrast to what would happen using cooler dialysis fluid. The contact with a warmer dialysis fluid could enhance the increase in blood flow (usually occurring at the start of the dialysis dwell), and hyperemia could have an important impact on solute transport [17].

Gross et al. [9] previously compared peritoneal urea clearance several times in one patient, comparing dialysate warmed at 37 °C with that infused at 20 °C. They reported a urea clearance that was 35% higher with the former compared to the latter. Unfortunately, their results are of scarce utility because a dialysate temperature of 20 °C is extremely lower than that used in clinical practice, in almost every setting. Moreover, their data come from seriate analysis in the same single patient. On the contrary, in an observational case-control study performed in Thailand, Indraprasit et al. [10] encountered no differences in peritoneal urea, creatinine and inulin clearances treating the patients with dialysate first at room temperature and then warmed up to 37 °C.

We performed a randomized study in order to ascertain the potential beneficial effects of higher dialysate temperature on peritoneal toxins clearances.

As can be judged by the results presented in Table 1, intervention and control groups were significantly different with respect to dialysate temperature at infusion and very well balanced for patients’ characteristics. Specifically, we encountered no differences in the proportions of males, diabetic patients and high-average peritoneal transporters, all factors that could have an influence on peritoneal clearances of toxins.

Treatment with dialysate at 37 °C failed to meet the primary end-point of superior peritoneal creatinine clearance compared to current clinical practice. Moreover, also urea peritoneal clearance, D/P for creatinine and urea, and peritoneal MTACs for urea and creatinine were not different between intervention and control groups.

We believe that the reasons for these relatively unexpected results can be related to the shortness of the temperature effects on microcirculation and to the influence of factors other than blood flow transport in small solutes peritoneal clearances.

Indeed, the vasoconstrictive effects of a lower temperature dialysate can be as brief as the first half hour of an exchange [17]. Since peritoneal dialysis temperature is rapidly equilibrated with body temperature, the potential effect of an initial lower dialysate temperature on toxin clearance is very difficult to ascertain in a 4 h PET. Moreover, blood flow is thought to have little (if any) effect on solute transport through the peritoneal membrane, and the effect of capillary exchange events on total transperitoneal transport is probably lower than expected due to the action of peritoneal interstitium, which is critical in the transport of small solutes [17]. Waniewski et al. [18] reported a 5–6 fold increase in the superficial peritoneal blood flow during the initial part of an ordinary peritoneal dialysis dwell in their mathematical PD model; nevertheless, the overall impact of this hyperemia on the measured creatinine flux was only approximately 60%, due to the impact of the interstitium, coupled in series with the capillaries, which critically modifies the overall peritoneal transport of small solutes. In addition, it is known that peritoneal dialysis fluids possess intrinsic vasodilator properties, mediated by endothelium-dependent mechanisms that primarily involve hyperosmolality [19, 20]. Vasodilation enhances small solutes and fluid transport, and typically occurs during the initial phase of a peritoneal dwell [21, 22], making difficult to ascertain a pure effect of dialysate temperature on peritoneal clearances.

In our study, patients in both groups reported a very low rate of side effects. Specifically, scores of the abdominal discomfort questionnaire were almost null, and did not differ between the groups. Warming peritoneal dialysate to 37 °C appeared to have no significant effects on blood pressure and body temperature during the PET. These data should reassure clinicians that peritoneal dialysate can be used at different temperatures without causing significant side effects or discomfort to patients.

Maintaining a high and constant dialysate temperature before infusion at every PD exchange is a time-consuming process that should be justified by a potential increase in depurative effects or the correction of symptoms that could appear with lower dialysate temperature. Also, in the era where portable and wearable PD devices are under development, it is very important to define whether a specific dialysate temperature should be addressed to avoid unnecessary challenges for the design that could hamper miniaturization.

In our study a higher dialysate temperature failed to provide evidence for superior peritoneal clearance of small molecules compared to common clinical practice at our center. We believe that any effort to rise dialysate temperature with the goal of increasing peritoneal depuration is not supported by evidence.

Future guidelines for PD should consider that evidence towards a recommendation for PD fluid warming to 37 °C is totally lacking.

Our study has some limitations. We acknowledge that the two ranges of dialysate temperature tested were relatively high and close to each other. As previously stated, the present study was intended as explorative; in addition, further lowering dialysate temperature in the control group was considered unfeasible due to ethical concerns and the theoretical risk of abdominal symptoms and hypothermia.

In addition, the study is monocentric and we included a relatively low number of PD patients for a single 4 h PET, limiting the generalization of our results in the long term. We enrolled a relatively low number of patients, for two main reasons. First, this was meant to be an explorative study, which could potentially have been expanded in case of problems with the randomization process. Second, the power analysis we performed before starting the study estimated a sample size of 7 patients per group (which we decided to increase to 9 per group) to achieve a statistical power of 95%, as stated above.

Furthermore, the lack of a cross-over design in our study could not account for possible interpatient differences generally existing among peritoneal functional behaviors. Nevertheless, we also consider that randomization of the present study adds strength to our findings.

Lastly, our study was not designed to assess the long-term effects of dialysate warming on peritoneal function.

Since some patients reported very little adherence to dialysate warming practice during their exchange routine, a further study designed to compare current practice with a lower dialysate temperature could be performed in the future.

## Conclusions

We report the results of a randomized controlled study assessing the effects of peritoneal dialysate temperature on peritoneal clearance of creatinine. Patients randomized to a peritoneal dialysate temperature of 37 °C did not show an increased creatinine peritoneal clearance during a 4 h PET when compared to patients treated with dialysate at uncontrolled temperature (around 32 °C). In addition, peritoneal clearance of urea and peritoneal transport of creatinine and urea did not differ between the groups. In both groups the incidence of abdominal discomfort was very low and dialysate temperature had no influence on blood pressure and body temperature. We believe that the potential increase in peritoneal microcirculatory blood flow related to the exposure to higher temperature (when compared with standard temperature) is too short to significantly enhance peritoneal small solute transport. The effect is probably further limited by the influence of the peritoneal interstitium. Clinicians should inform patients about the lack of benefit of warming peritoneal dialysate to 37 °C and future PD guidelines should not reinforce this recommendation.

## Supplementary Information

**Additional file 1.**

## Data Availability

All data were stored in the repository of the Nephrology Unit, Policlinico di Modena, Azienda Ospedaliero Universitaria di Modena, and are available upon request.

## Abbreviations

PD: Peritoneal dialysis
CAPD: Continuous ambulatory peritoneal dialysis
GDPs: Glucose degradation products
D/P: Dialysate to plasma ratio
MTAC: Mass transfer area coefficients
PET: Peritoneal equilibration test
